# Comparative Study of Structure-Property Relationships in Polymer Networks Based on Bis-GMA, TEGDMA and Various Urethane-Dimethacrylates

**DOI:** 10.3390/ma8031230

**Published:** 2015-03-19

**Authors:** Izabela Barszczewska-Rybarek, Sebastian Jurczyk

**Affiliations:** 1 Department of Physical Chemistry and Technology of Polymers, Silesian University of Technology, Gliwice 44-100, Poland; 2 Paint and Plastics Department, Institute for Engineering of Polymer Materials and Dyes, Gliwice 44-100, Poland; E-Mail: s.jurczyk@impib.pl

**Keywords:** dental resin, urethane-dimethacrylate, Bis-GMA, TEGDMA, microgel agglomerates, degree of conversion, mechanical properties, morphology, fracture behavior, SEM

## Abstract

The effect of various dimethacrylates on the structure and properties of homo- and copolymer networks was studied. The 2,2-bis-[4-(2-hydroxy-3-methacryloyloxypropoxy)phenyl]-propane) (Bis-GMA), triethylene glycol dimethacrylate (TEGDMA) and 1,6-bis-(methacryloyloxy-2-ethoxycarbonylamino)-2,4,4-trimethylhexane (HEMA/TMDI), all popular in dentistry, as well as five urethane-dimethacrylate (UDMA) alternatives of HEMA/TMDI were used as monomers. UDMAs were obtained from mono-, di- and tri(ethylene glycol) monomethacrylates and various commercial diisocyanates. The chemical structure, degree of conversion (*DC*) and scanning electron microscopy (SEM) fracture morphology were related to the mechanical properties of the polymers: flexural strength and modulus, hardness, as well as impact strength. Impact resistance was widely discussed, being lower than expected in the case of poly(UDMA)s. It was caused by the heterogeneous morphology of these polymers and only moderate strength of hydrogen bonds between urethane groups, which was not high enough to withstand high impact energy. Bis-GMA, despite having the highest polymer morphological heterogeneity, ensured fair impact resistance, due to having the strongest hydrogen bonds between hydroxyl groups. The TEGDMA homopolymer, despite being heterogeneous, produced the smoothest morphology, which resulted in the lowest brittleness. The UDMA monomer, having diethylene glycol monomethacrylate wings and the isophorone core, could be the most suitable HEMA/TMDI alternative. Its copolymer with Bis-GMA and TEGDMA had improved *DC* as well as all the mechanical properties.

## 1. Introduction

**Scheme 1:**
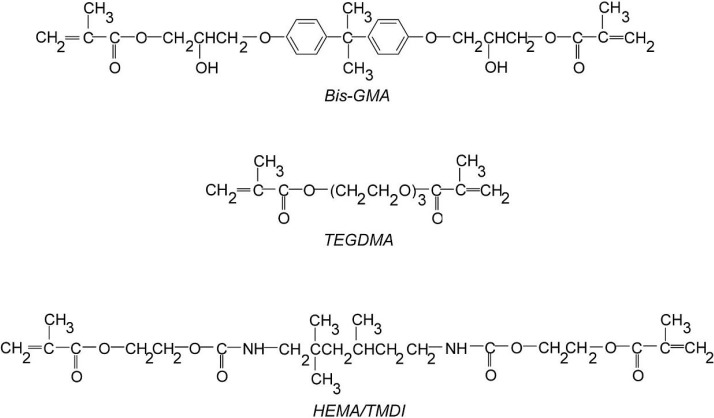
Monomers commonly used in dental practice.

Current dental composites consist of three essential components: a crosslinked polymer matrix, a high volume fraction of inorganic filler and a coupling agent added to ensure matrix-filler adhesion. Within composite resins, the 2,2-bis-[4-(2-hydroxy-3-methacryloyloxypropoxy)phenyl]-propane) (Bis-GMA), 1,6-bis-(methacryloyloxy-2-ethoxycarbonylamino)-2,4,4-trimethylhexane (HEMA/TMDI) and triethylene glycol dimethacrylate (TEGDMA) are the ones mostly applied in dental practice (Scheme 1). Bis-GMA is the most commonly used [1]. The stiff molecular structure and hydroxyl groups of Bis-GMA are responsible for low polymerization shrinkage, high polymer modulus, and desirable adhesion to tooth enamel. Regrettably, they cause extremely high resin viscosity, residual unsaturation in the polymer as well as water uptake. The reduction in viscosity and the increase in the degree of conversion are generally achieved by the addition of reactive diluents. TEGDMA is usually added for this purpose, in amounts from 20 wt% to 50 wt% [1,2,3,4]. On the other hand, TEGDMA increases polymerization shrinkage and matrix water sorption [1,2,5]. Alternative dental formulations contain the urethane-dimethacrylate monomer—HEMA/TMDI. The advantage of HEMA/TMDI is its lower viscosity, when compared to Bis-GMA. Moreover, urethane linkage can form strong hydrogen bonds and thus improve both the durability of the composite’s matrix as well as its bonding to the tooth structure. HEMA/TMDI is used alone or in a combination with Bis-GMA and TEGDMA [1,2,3,4].

In fact, HEMA/TMDI (Scheme 1) represents a wide family of urethane-dimethacrylate monomers (UDMA). Their chemical structures can easily be tailored through an appropriate choice of the core and wing segments, resulting in diversity of monomers and corresponding polymers with a wide range of chemical and physico-mechanical properties.

**Scheme 2:**
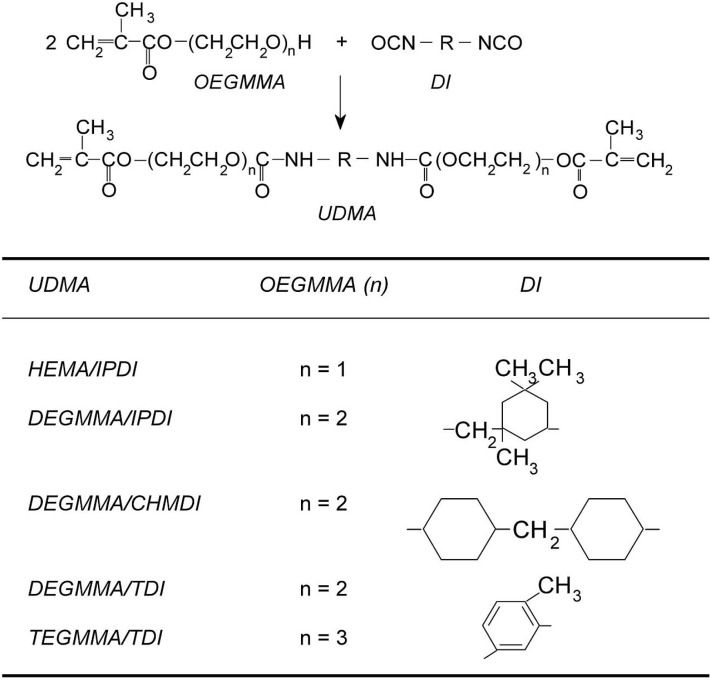
The alternative UDMA monomers used in this work.

In the previous paper, twenty four UDMA monomers, being the structural analogues of HEMA/TMDI, and their homopolymers were characterized [6]. The monomer cores derived from six commercially available diisocyanates (DI): aliphatic–HMDI and TMDI, cycloaliphatic–IPDI and CHMDI, aromatic–TDI and MDI. The wing structures originated from oligo(ethylene glycols) monomethacrylates (OEGMMA), consisting of up to four oxyethylene units in the oligooxyethylene chains [6]. Based on the results of those studies, HEMA/IPDI, DEGMMA/IPDI, DEGMMA/CHMDI, DEGMMA/TDI, as well as TEGMMA/TDI were selected for further testing of structure-property relationships in dimethacrylate polymer networks (Scheme 2).

The general purpose of this work was to check if these five monomers could improve the polymer network structure and mechanical properties, when copolymerized. In order to achieve this aim two compositions, commonly utilized in dentistry, consisting of Bis-GMA and TEGDMA as well as Bis-GMA, TEGDMA and HEMA/TMDI, were modified by replacing, respectively Bis-GMA with HEMA/IPDI as well as HEMA/TMDI with all five UDMAs. Cured and unfilled adhesives were characterized by the monomer chemical structure and hydrogen bonding as well as the polymer degree of conversion, SEM morphology, flexural modulus, flexural strength, hardness, and impact resistance.

Understanding the influence of various dimethacrylate monomers on the structure and mechanical properties of their homo- and copolymer networks can give inspiration for designing new and more efficient copolymer formulations.

## 2. Results and Discussion

In this work, five alternative UDMA monomers: HEMA/IPDI, DEGMMA/IPDI, DEGMMA/CHMDI, DEGMMA/TDI and TEGMMA/TDI (Scheme 2) were synthesized in the addition reaction of oligo(ethylene glycols) monomethacrylates (OEGMMA). They consist of one to three oxyethylene units in the oligooxyethylene chains and three various cores derived from commercial diisocyanates (DI): CHMDI (cycloaliphatic), IPDI (cycloaliphatic) and TDI (aromatic). Bis-GMA, HEMA/TMDI and TEGDMA were also synthesized, as popular dental resins (Scheme 1). Basic properties of monomers used in this study are presented in Table 1.

**Table 1:** The properties of used dimethacrylates: molecular weight (*MW*), concentration of double bonds (*X_DB_*), viscosity (η).

Monomer	*MW* (g/mol)	*X_DB_* (mol/kg)	η (Pa·s)
HEMA/TMDI	470.6	4.25	6.22 ^a^
HEMA/IPDI	482.5	4.14	12.33 ^a^
DEGMMA/IPDI	570.7	3.50	8.80 ^a^
DEGMMA/CHMDI	610.8	3.27	16.66 ^a^
DEGMMA/TDI	522.6	3.82	13.75 ^a^
TEGMMA/TDI	610.7	3.27	10.27 ^a^
Bis-GMA	512.6	3.90	1200 ^b^
TEGDMA	286.3	6.99	0.011 ^b^

^a^ As cited in Ref. [6]; ^b^ As cited in Ref. [7].

Two groups of monomer formulations were prepared and photopolymerized, utilizing a camphorquinone/tertiary amine photo-initiating system. In the first group, Bis-GMA and HEMA/IPDI were copolymerized with TEGDMA, in 80:20 and 60:40 weight ratios. In the second group, each of the UDMA monomers was copolymerized with Bis-GMA and TEGDMA, in a 38:42:20 weight ratio. Finally, several copolymer networks were obtained, their compositions are shown in Table 2.

**Table 2:** The copolymer names and compositions.

The Copolymer Name	The Sample Composition					
	UDMA		Bis-GMA		TEGDMA	
	wt%	mol%	wt%	mol%	wt%	mol%
Bis-GMA–TEGDMA (60:40)	-	-	60	45.6	40	54.4
Bis-GMA–TEGDMA (80:20)	-	-	80	69.1	20	30.9
HEMA/IPDI–TEGDMA (60:40)	60	47.1	-	-	40	52.9
HEMA/IPDI–TEGDMA (80:20)	80	70.4	-	-	20	29.6
HEMA/TMDI–Bis-GMA–TEGDMA	38	34.7	42	35.3	20	30.0
HEMA/IPDI–Bis-GMA–TEGDMA	38	34.2	42	35.5	20	30.3
DEGMMA/IPDI–Bis-GMA–TEGDMA	38	30.5	42	37.5	20	32.0
DEGMMA/CHMDI–Bis-GMA–TEGDMA	38	29.1	42	38.3	20	32.6
DEGMMA/TDI–Bis-GMA–TEGDMA	38	32.4	42	36.5	20	31.1
TEGMMA/TDI–Bis-GMA–TEGDMA	38	29.1	42	38.3	20	32.6

### 2.1. HEMA/IPDI as Bis-GMA Substitute in Copolymers with TEGDMA

The substitution of Bis-GMA with HEMA/IPDI, in formulations with TEGDMA, provided the first type of copolymers. This was achieved by mixing HEMA/IPDI as well as Bis-GMA with TEGDMA, in 80:20 and 60:40 wt% ratios, and photopolymerization. For comparative purposes, HEMA/IPDI, Bis-GMAand TEGDMA homopolymers were produced.

**Figure 1:**
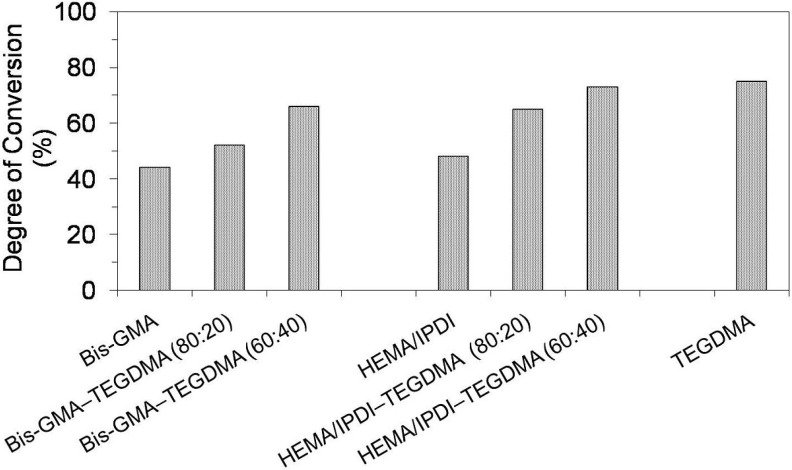
The degree of conversion in Bis-GMA, HEMA/IPDI and TEGDMA homopolymers as well as Bis-GMA–TEGDMA and HEMA/IPDI–TEGDMA copolymers.

Figure 1 presents results for the degree of conversion (*DC*) in homopolymer and copolymer networks. The *DC* is crucial for understanding the physico-mechanical behavior of the polymer network [8,9]. When IR spectroscopy is applied to investigate the *DC* in poly(dimethacrylate)s, the internal band ratio method with stretching vibrations of the aromatic ring, as a reference, is the most frequently used [3,8]. However, due to the lack of aromatic moieties in the majority of examined compositions, the carbonyl vibrations peak was chosen as an internal standard to calculate the *DC* in polymers. This method is often used, when monomers have no aromatic rings [8,10]. However, it might produce a lower *DC* than the method using an aromatic band as a standard, especially for monomers with a greater stiffness [8].

As can be seen in Figure 1, the homopolymers had lower *DC* values than copolymers. The lowest *DC* in the Bis-GMA homopolymer, being of 40%, can be interpreted by low elasticity and mobility of the monomer. The Bis-GMA molecule is long and spacious; it has two *p*-phenylene rings and two hydroxyl groups, involved in strong intermolecular hydrogen bonds. These features cause a dramatic increase in viscosity (1200 Pa·s, Table 1) and a decrease in the *DC* [3]. HEMA/IPDI, when compared to Bis-GMA, is 99% less viscous (12 Pa·s, Table 1) [6]. Additionally, two elastic urethane linkages may offer an alternative polymerization path, causing chain transfer reactions, which increase the mobility of radical sites in the network [3]. With regard to this aspect, HEMA/IPDI might be expected to polymerize to a more suitable *DC*. However, the factor of high molecular stiffness, resulting from the cycloaliphatic IPDI core, seems to explain the relatively low *DC* in the homopolymer, which is 48%.

As far as Figure 1 is concerned, the degree of conversion in both groups of copolymers increased with increasing TEGDMA content. This behavior can be associated with two effects: the resin viscosity and the chemical structure allowing for molecular mobility [11,12]. The small size of the TEGDMA molecule and the high concentration of double bonds ensure a close proximity between radical sites, whereas its high flexibility and the absence of the hydrogen bond proton donor incur low resin viscosity (of 0.011 Pa·s [7], Table 1). The degree of conversion in HEMA/IPDI copolymers (65% and 73%) was higher than the *DC* in corresponding Bis-GMA copolymers (52% and 66%). It could be generally concluded, that the copolymerization of dimethacrylates having hydrogen bond proton donors (UDMA and Bis-GMA) with TEGDMA, having only proton acceptors, improved the degree of conversion within the network. Additionally, it worked more effectively in systems composed of HEMA/IPDI, being the urethane-dimethacrylate, rather than in Bis-GMA systems. These results were in agreement with the findings from other studies, performed on similar dimethacrylate systems [11,12,13].

**Figure 2:**
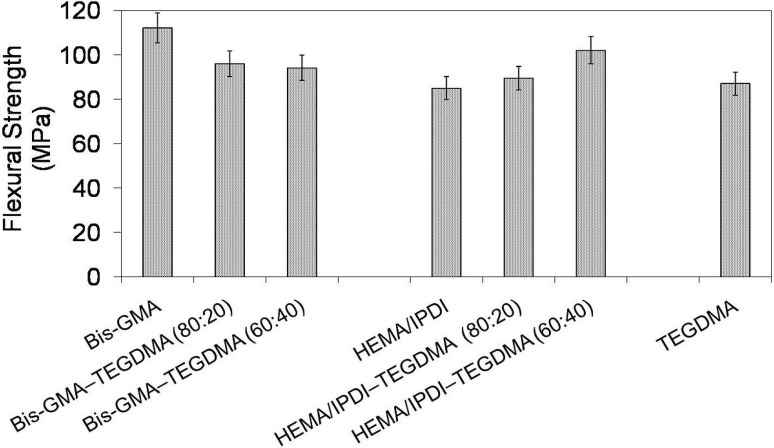
The flexural strength of Bis-GMA, HEMA/IPDI and TEGDMA homopolymers as well as Bis-GMA–TEGDMA and HEMA/IPDI–TEGDMA copolymers.

In Figure 2 the relationship between flexural strength and polymer composition is depicted. The mechanical strength of investigated homopolymers ranges from 85 to 112 MPa and follows this order: σ_poly(HEMA/IPDI)_ < σ_poly(TEGDMA)_ < σ_poly(Bis-GMA)_. The flexural strength of the Bis-GMA–TEGDMA copolymers slightly decreased as the TEGDMA content increased. In contrast, the flexural strength of the HEMA/IPDI–TEGDMA copolymers increased as the TEGDMA content was raised. The HEMA/IPDI–TEGDMA (60:40) network had the highest flexural strength among the copolymers of this group, having a value of 102 MPa.

**Figure 3:**
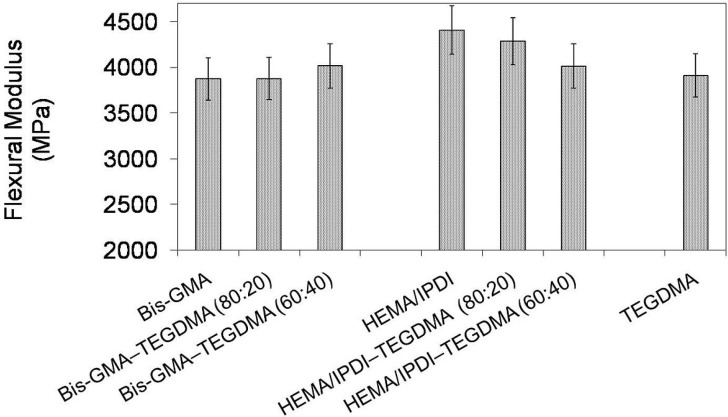
The flexural modulus of Bis-GMA, HEMA/IPDI and TEGDMA homopolymers as well as Bis-GMA–TEGDMA and HEMA/IPDI–TEGDMA copolymers.

In Figure 3 the relationship between flexural modulus and polymer compositions is depicted. One can observe the modulus of investigated homopolymers in the following order: *E*_poly(Bis-GMA)_ < *E*_poly(TEGDMA)_ < *E*_poly(HEMA/IPDI)_ and in the range from 3872 to 4406 MPa. The modulus of the Bis-GMA–TEGDMA copolymers was raised with increasing TEGDMA fraction. In contrast, the modulus of the HEMA/IPDI–TEGDMA copolymers decreased with increasing TEGDMA content. The Bis-GMA–TEGDMA (80:20) copolymer had the lowest modulus in this group, of 3876 MPa, whereas the analogous HEMA/IPDI–TEGDMA (80:20) copolymer had the highest *E*, of 4283 MPa.

In order to explain these contrary flexural property-composition relationships, for each group of copolymers, a different approach should be implemented. In the Bis-GMA–TEGDMA (80:20) system, Bis-GMA still seriously limited the system mobility and sterically isolated methacrylate groups against polymerization. The TEGDMA content was not high enough to significantly improve the degree of conversion and as a consequence, the modulus remained almost unchanged. On the other hand, TEGDMA influenced the flexural strength by decreasing its value. When the TEGDMA content was raised to 40 wt%, the degree of conversion increased from 52% to 66%. The improved copolymerization scale caused the decrease in the copolymer elasticity, thereby decreasing the strain at the break, from 96 to 94 MPa, and hence increasing the modulus, from 3876 to 4014 MPa. HEMA/IPDI, if compared to Bis-GMA, produced homopolymer networks of a higher degree of conversion and higher modulus. After copolymerization with TEGDMA, an increase in elasticity was observed, despite an increase in the *DC*. The reason for this could be due to the TEGDMA elasticity. The homopolymer of TEGDMA, characterized by the modulus of 3910 MPa, is more elastic than that of HEMA/IPDI. Consequently, the decrease in flexural modulus and the increase of flexural strength, proportional to the TEGDMA content, were observed.

**Figure 4:**
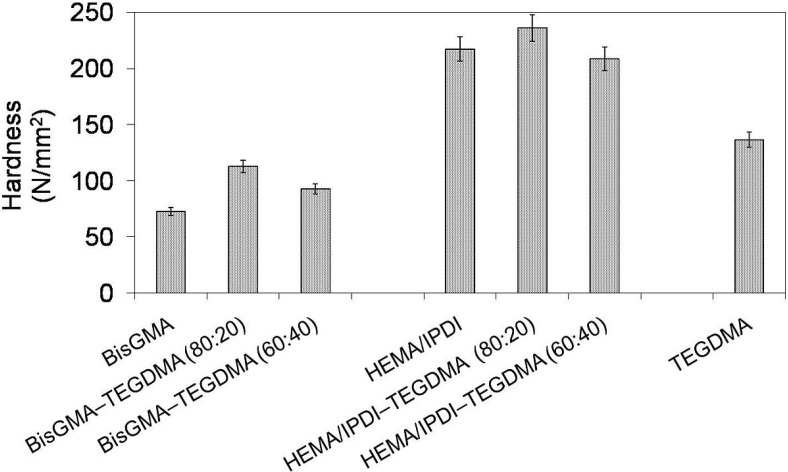
The Brinell hardness of Bis-GMA, HEMA/IPDI and TEGDMA homopolymers as well as Bis-GMA–TEGDMA and HEMA/IPDI–TEGDMA copolymers.

In Figure 4 the relationship between Brinell hardness (*HB*) and monomer composition is depicted. One can note that the hardness of investigated homopolymers follows this order: *HB*_poly(Bis-GMA)_ < *HB*_poly(TEGDMA)_ < *HB*_poly(HEMA/IPDI)_ and ranges from 73 to 217 N/mm^2^. The hardness of copolymers, after the initial growth, dropped with increase of TEGDMA content. Copolymers of HEMA/IPDI were characterized by significantly higher hardness (236 and 208 N/mm^2^) than copolymers of Bis-GMA (113 and 93 N/mm^2^). This behavior implies a strong influence of the degree of conversion on *HB*. The initial increase in *HB* resulted from the *DC* increase. The consequent decrease in *HB*, accompanied by an increase in the degree of conversion, was affected by the TEGDMA structure.

**Figure 5:**
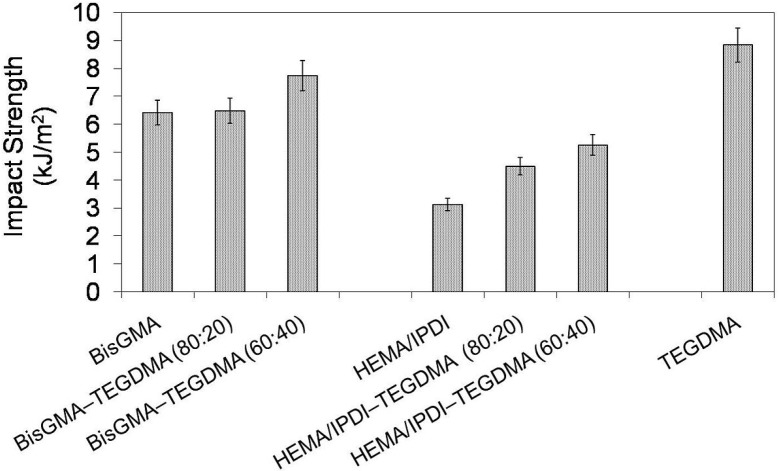
The impact resistance of Bis-GMA, HEMA/IPDI and TEGDMA homopolymers as well as Bis-GMA–TEGDMA and HEMA/IPDI–TEGDMA copolymers.

In Figure 5 the relationship between impact strength and monomer composition is depicted. It may be seen that the HEMA/IPDI homopolymer had the lowest impact strength of 3.1 kJ/m^2^, poly(Bis-GMA) had 6.4 kJ/m^2^ and poly(TEGDMA) had the highest, of 8.8 kJ/m^2^. Thereby, the order: *a_n_*_poly(UDMA)_ < *a_n_*_poly(Bis-GMA)_ < *a_n_*_poly(TEGDMA)_, which was found in previous studies, was confirmed [14]. The copolymers, in general, had better resistance to cracking than homopolymers. Copolymers of HEMA/IPDI were characterized by higher brittleness (*a_n_* = 4.5 and 5.3 kJ/m^2^) than copolymers of Bis-GMA (*a_n_* = 6.5 and 7.7 kJ/m^2^). The higher the TEGDMA content, the higher the impact resistance determined.

The impact resistance of poly(urethane-dimethacrylate)s, due to its lower than expected values, remains a subject of great concern [9,14,15]. As shown in earlier works, the fracture behavior of dimethacrylate polymer networks is a complex function of the monomer size and stiffness, the degree of conversion and the polymer morphology [9,14,15]. Homo- and copolymerizations of UDMA, Bis-GMA and TEGDMA lead to the formation of strongly heterogeneous morphologies, composed of microgel agglomerates of different sizes and crosslink densities [9,14,16,17,18,19]. Previous XRPD studies on these homopolymers [14] as well as on their acrylate analogues [9] have shown that monomers having groups involved in hydrogen bonds with both the proton donor as well as with the acceptor, form more massive clusters than TEGDMA, having only the proton acceptor. Consequently, the homopolymers of UDMA and Bis-GMA were more brittle than the TEGDMA homopolymer.

Impact strength of the Bis-GMA homopolymer was shown to be twice as high as the impact strength of the HEMA/IPDI homopolymer. This behavior does not seem to be caused by the degree of conversion or microgel agglomerate dimensions. The latter network had higher crosslink density, resulting from a higher concentration of double bonds (Table 1) as well as a higher degree of conversion (Figure 1). Both monomers form strong hydrogen bonds and they organize themselves into microgel agglomerates of similar sizes through polymerization. The reason for the weak poly(UDMA) impact resistance might be the overall crosslink density in a less crosslinked matrix, in which highly crosslinked microgels are embedded. In the HEMA/IPDI as well as the Bis-GMA polymer networks, two kinds of crosslinks can be distinguished: permanent, covalent crosslinks and physical crosslinks, resulting from hydrogen bonds. The NH···N and NH···O hydrogen bonds, present in the UDMA system, are weaker than the O–H···O hydrogen bonds, present in the Bis-GMA (Table 3) [11,20]. Hydrogen bonds between urethane groups are most probably not strong enough to bond microgel agglomerates in poly(UDMA) and to withstand high impact energies. The poly(Bis-GMA), where microgel agglomerates are even bigger, was shown to be tougher, since physical crosslinks are stronger. Finally, it could be concluded that impact resistance of poly(dimethacrylate)s depends on the following factors. When the polymer network morphology is less heterogeneous and consists of small clusters the impact resistance is good. When the network morphology is more heterogeneous, with massive clusters, the strength of hydrogen bonds determines the crack resistance. This situation is observed for polymer networks produced from stiff monomers, having groups involved in hydrogen bonding. The higher the strength of physical bonding, the higher the impact strength observed.

**Table 3:** Types of hydrogen bonds in studied dimethacrylates and corresponding energies [20].

Type of Hydrogen Bond	Energy (kJ/mol)
O–H···N	29
O–H···O	21
N–H···N	13
N–H···O	8

**Figure 6:**
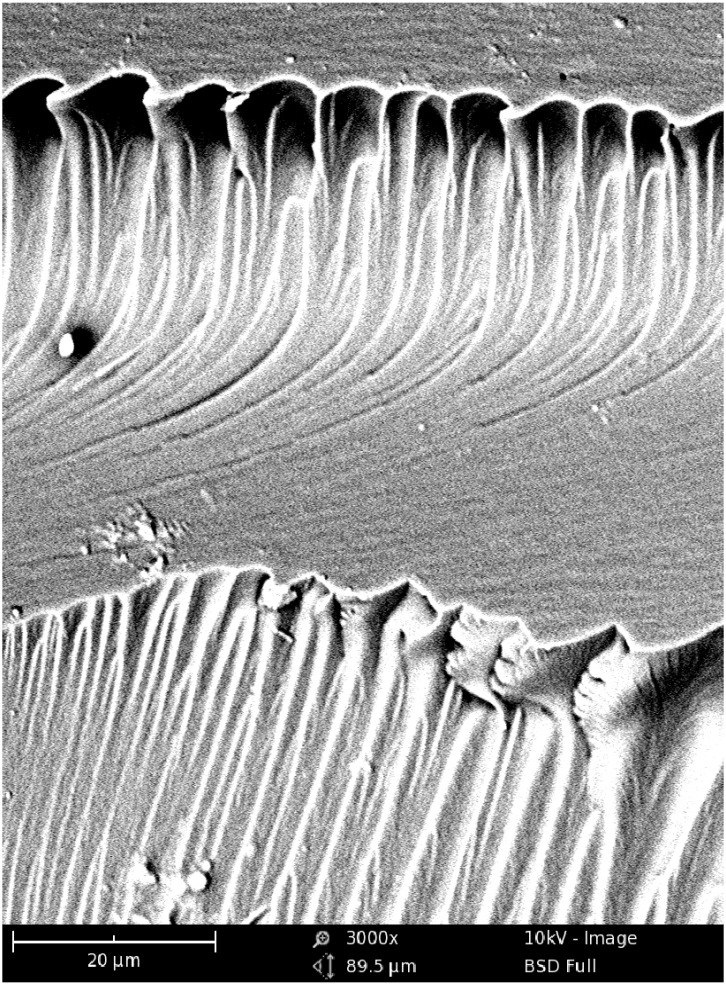
The SEM image of the Bis-GMA–TEGDMA (80:20) fractured surface; a scale bar represents 20 μm.

**Figure 7:**
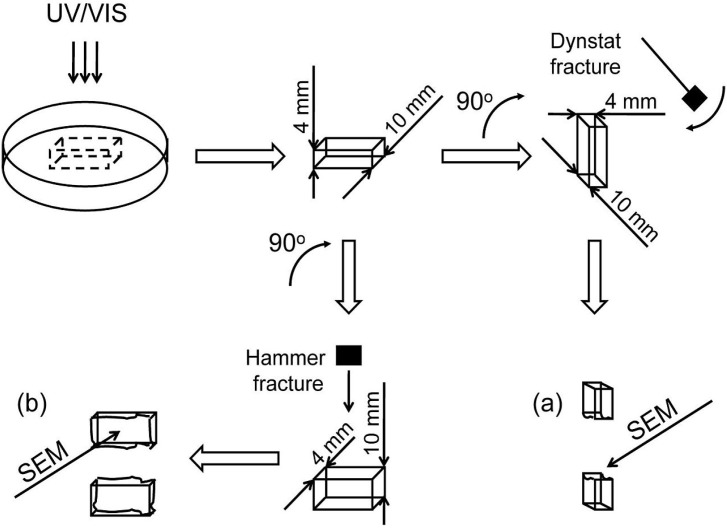
The representation of the fracture directions: (**a**) from impact tests, fracture surface was parallel to the direction of irradiation; (**b**) made with a hammer, fracture surface was perpendicular to the direction of irradiation.

**Figure 8:**
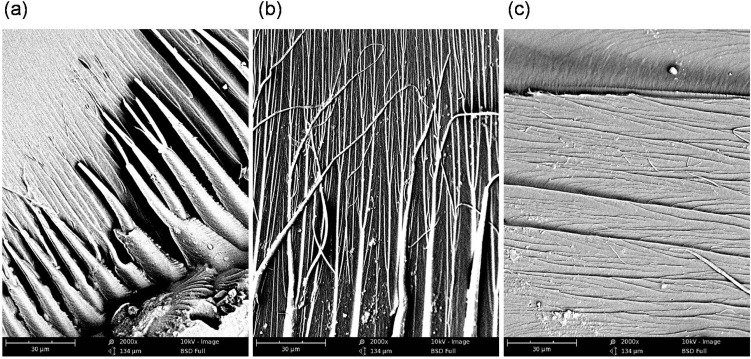
The SEM images of: (**a**) and (**b**) the Bis-GMA–TEGDMA (80:20); (**c**) HEMA/IPDI–TEGDMA (80:20) fractured surfaces; a scale bars represents 30 μm.

In this study, SEM was used for imaging the internal morphologies of polymer networks produced from Bis-GMA, HEMA/IPDI and TEGDMA. Figure 6 shows the exemplary micrograph of the Bis-GMA–TEGDMA (80:20) sample, fractured in impact tests. The observed surface was parallel to the direction of the UV/VIS irradiation (Figure 7a). The SEM image revealed patterns, suggesting a unidirectional orientation of microgel agglomerates, perpendicular to this fracture, *i.e.*, perpendicular to the light direction. This finding was confirmed by SEM analysis of the fractures, performed during controlled crack propagation, presenting surfaces perpendicular to the direction of irradiation (Figure 8, Figure 7b). The copolymers revealed a morphology, consisting of nodular objects arranged in a regular array, perpendicular to the UV/VIS light. The Bis-GMA copolymers revealed sharper-edged morphological objects in comparison to the HEMA/IPDI copolymers. It suggests that the chemical crosslinking and the agglomerate dimensions determine the fracture pattern. However, impact strength values are dependent on the overall energy of interactions between clusters in poly(dimethacrylate)s, coming from chemical and physical crosslinks.

### 2.2. Alternative UDMA Monomers as HEMA/TMDI Replacement in Copolymers with Bis-GMA and TEGDMA

The second group of copolymers was tested for the influence of various UDMA monomers on the properties of networks, produced by their copolymerization with Bis-GMA and TEGDMA. This was achieved by mixing HEMA/IPDI, DEGMMA/IPDI, DEGMMA/CHMDI, DEGMMA/TDI, as well as TEGMMA/TDI with Bis-GMA and TEGDMA, in a 38:42:20 wt% ratio, and photopolymerized. The popular dentistry copolymer of HEMA/TMDI–Bis-GMA–TEGDMA (38:42:20) was produced as the precursor. For comparative purposes, the homopolymers of all UDMAs were obtained.

**Figure 9:**
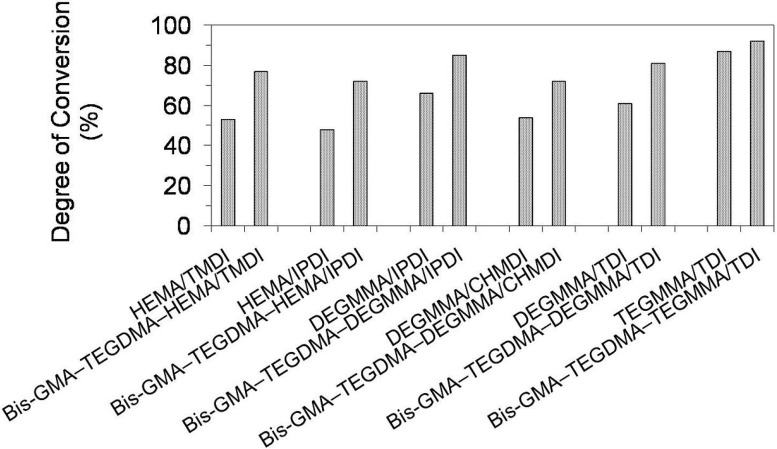
The degree of conversion in homopolymers and copolymers composed of UDMA, Bis-GMA and TEGDMA.

Figure 9 presents results for the degree of conversion (*DC*) in homopolymer and copolymer networks. The *DC* in homopolymers ranged from 48% to 87% and followed the order: *DC*_poly(HEMA/IPDI)_ < *DC*_poly(HEMA/TMDI)_ < *DC*_poly(DEGMMA/CHMDI)_ < *DC*_poly(DEGMMA/TDI)_ < *DC*_poly(DEGMMA/IPDI)_ < *DC*_poly(TEGMMA/TDI)_. As can be seen, increasing oligooxyethylene chain length caused an increase of the UDMA elasticity and mobility, giving rise to an increase in the *DC*. The diisocyanate chemical character also influenced the homopolymer *DC*, which increased in the following way: cycloaliphatic symmetrical (CHMDI) < aromatic asymmetrical (TDI) < cycloaliphatic asymmetrical (IPDI) < aliphatic (TMDI). The lowest *DC* in the HEMA/IPDI homopolymer, of 48%, can be explained by the spacious and stiff cycloaliphatic ring, asymmetrically substituted with two short HEMA wings. HEMA/TMDI, which is sometimes used alone in dental formulations [4] when homopolymerized, achieved a *DC* of 53% slightly exceeding 50%. The *DC* in homopolymers, having DEGMMA was found within the range of 54%–66%, whereas in poly(TEGMMA/TDI) it equaled 87%.

Copolymerization resulted in an increase in the degree of conversion in polymer networks, when compared to UDMA homopolymers. The *DC* in copolymers ranged from 72% to 92%. The differences in *DC*s between corresponding homo- and copolymers decreased with increasing oligooxyethylene chain length. The increases of around 50% in the *DC* were recorded for UDMAs, having HEMA, of around 30% for those having DEGMMA, and 8% in the case of TEGDMA/TDI. It can be concluded, that copolymerization is beneficial for the structural heterogeneity of dimethacrylate networks from the perspective of the degree of conversion.

**Figure 10:**
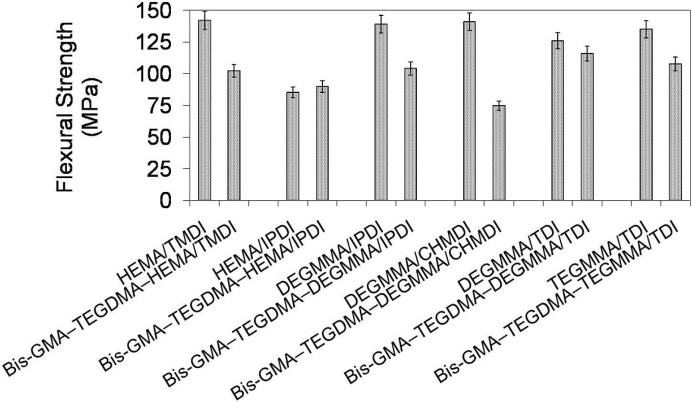
The flexural strength of homopolymers and copolymers composed of UDMA, Bis-GMA and TEGDMA.

In Figure 10 the relationship between flexural strength and polymer composition is depicted. It is seen, that flexural strengths of the investigated homopolymers ranged from 85 to 142 MPa and followed the order: σ_poly(HEMA/IPDI)_ < σ_poly(DEGMMA/TDI)_ < σ_poly(TEGMMA/TDI)_ < σ_poly(DEGMMA/IPDI)_ < σ_poly(DEGMMA/CHMDI)_ = σ_poly(HEMATMDI)_. The flexural strengths of their corresponding copolymers were lower, except HEMA/IPDI, and ranged from 75 to 116 MPa, following this order: σ_poly(DEGMMA/CHMDI)_ < σ_poly(HEMA/IPDI)_ < σ_poly(HEMATMDI)_ < σ_poly(DEGMMA/IPDI)_ < σ_poly(DEGMMA/TDI)_ < σ_poly(TEGMMA/TDI)_. The HEMA/IPDI copolymer had a flexural strength of 90 MPa, which was slightly higher than σ of the homopolymer. This could be explained by the TEGDMA and Bis-GMA influence. The flexural strength values of poly(TEGDMA) (87 MPa), and poly(Bis-GMA) (112 MPa) were higher than σ corresponding to poly(HEMA/IPDI) (85 MPa) and lower than σ corresponding to the remaining UDMA homopolymers, starting from 126 MPa. The copolymers of the following alternative UDMA monomers: DEGMMA/IPDI, DEGMMA/TDI and TEGMMA/TDI had a higher flexural strength than the HEMA/TMDI–Bis-GMA–TEGDMA copolymer.

**Figure 11:**
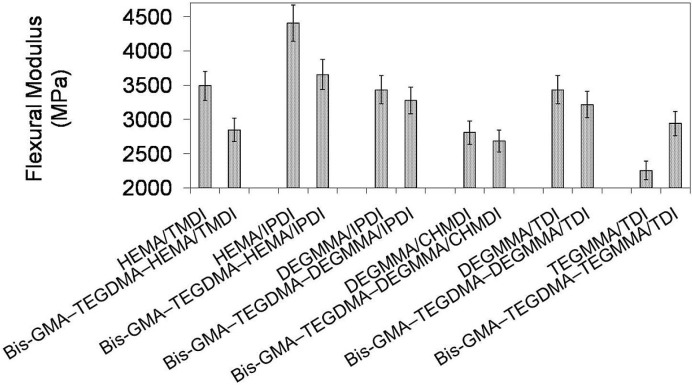
The flexural modulus of homopolymers and copolymers composed of UDMA, Bis-GMA and TEGDMA.

In Figure 11 the relationship between flexural modulus and polymer compositions is depicted. One can observe that the modulus of investigated homopolymers followed the order: *E*_poly(TEGMMA/TDI)_ < *E*_poly(DEGMMA/CHMDI)_ < *E*_poly(DEGMMA/TDI)_ = *E*_poly(DEGMMA/IPDI)_ < *E*_poly(HEMA/TMDI)_ < *E*_poly(HEMA/IPDI)_ and ranged from 2252 to 4406 MPa. The modulus of copolymers ranged from 2681 to 3653 MPa and increased in this order: *E*_poly(DEGMMA/CHMDI)_ < *E*_poly(HEMA/TMDI)_ < *E*_poly(TEGMMA/TDI)_ < *E*_poly(DEGMMA/TDI)_ < *E*_poly(DEGMMA/IPDI)_ < *E*_poly(HEMA/IPDI)_. The modulus of the copolymer was always lower than *E* of the corresponding UDMA homopolymer, except TEGMMA/TDI. In this case, the increase in modulus resulted from significantly higher stiffness of Bis-GMA and TEGDMA than TEGMMA/TDI. The copolymers of the following alternative UDMA monomers: HEMA/IPDI, DEGMMA/IPDI, DEGMMA/TDI and TEGMMA/TDI had a modulus higher than the HEMA/TMDI–Bis-GMA–TEGDMA copolymer.

**Figure 12:**
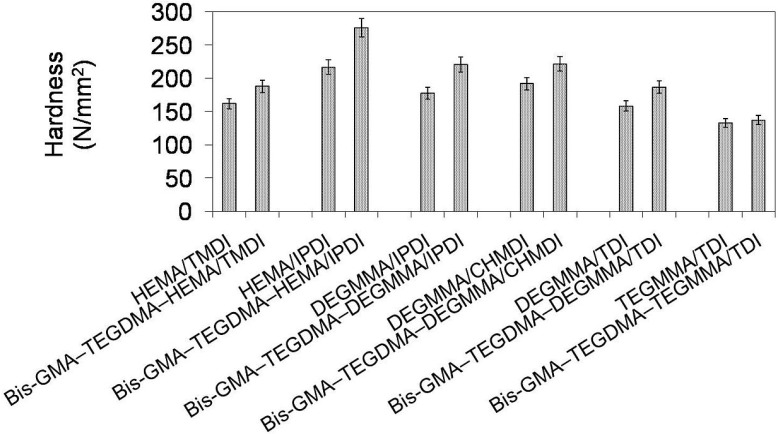
The Brinell hardness of homopolymers and copolymers composed of UDMA, Bis-GMA, and TEGDMA.

In Figure 12 the relationship between Brinell hardness and monomer composition is depicted. One can note that the hardness of homopolymers as well as copolymers followed the same order: *HB*_poly(TEGMMA/TDI)_ < *HB*_poly(DEGMMA/TDI)_ < *HB*_poly(HEMA/TMDI)_ < *HB*_poly(DEGMMA/IPDI)_ < *HB*_poly(DEGMMA/CHMDI)_ < *HB*_poly(HEMA/IPDI)_. Hardness values of homopolymers ranged from 133 to 217 N/mm^2^, whereas values of corresponding copolymers were higher and ranged from 137 to 276 N/mm^2^. These increases might be explained by the increases in the degree of conversion. HEMA/IPDI demonstrated a very positive effect on the copolymer hardness, which was the highest among those studied. DEGMMA/IPDI and DEGMMA/CHMDI also improved the copolymer hardness, in relation to the HEMA/TMDI–Bis-GMA–TEGDMA copolymer.

**Figure 13:**
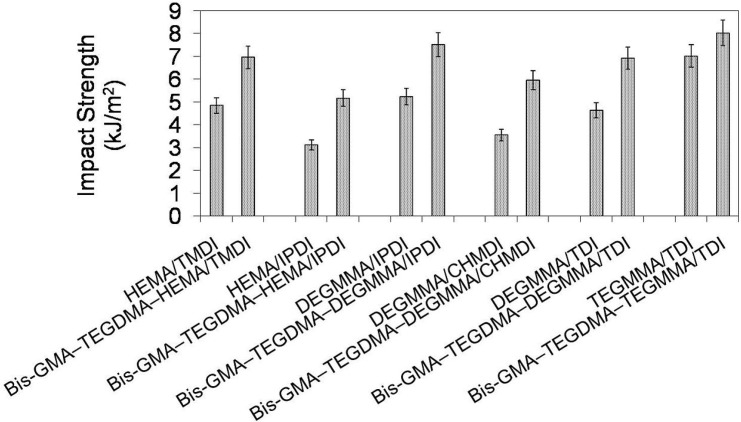
The impact strength of homopolymers and copolymers composed of UDMA, Bis-GMA, and TEGDMA.

In Figure 13 the relationship between impact strength and monomer composition is depicted. It may be seen that the impact resistance of homopolymers as well as copolymers followed the same order: *a_n_*_poly(HEMA/IPDI)_ < *a_n_*_poly(DEGMMA/CHMDI)_ < *a_n_*_poly(DEGMMA/TDI)_ < *a_n_*_poly(HEMA/TMDI)_ < *a_n_*_poly(DEGMMA/IPDI)_ < *a_n_*_poly(TEGMMA/TDI)_. Impact strength for UDMA homopolymers could be found within 3.12–7.02 kJ/m^2^, whereas for corresponding copolymers from 5.17 to 8.03 kJ/m^2^. The improvement of fracture resistance resulting from copolymerization can be explained by the TEGDMA properties. The TEGDMA homopolymer had the highest impact strength, of 8.83 kJ/m^2^ among the polymers studied here. When comparing the HEMA/TMDI–Bis-GMA–TEGDMA copolymer to the copolymers with alternative UDMAs, the improvement of impact resistance was achieved when DEGMMA/IPDI and TEGMMA/TDI were used.

## 3. Experimental Section

### 3.1. Materials

Urethane-dimethacrylate monomers (UDMA) were synthesized from oligo(ethylene glycols) monomethacrylates (OEGMMA) and diisocyanates (DI) according to the procedure previously reported [21,22]. OEGMMA: DEGMMA and TEGMMA were obtained through a trans-esterification reaction of methyl methacrylate (MMA, Acros, Geel, Belgium) with the corresponding glycols: diethylene (DEG, Acros, Geel, Belgium) and triethylene (TEG, Acros, Geel, Belgium), according to the procedure previously described [21,22]. The Bis-GMA monomer was synthesized from 2,2-Bis[4-(2,3-epoxypropoxy)phenyl]propane (BADGE, DER 330, The Dow Chemical Company, Midland, MI, USA, EV = 0.57 mol/100 g epoxy groups), methacrylic acid (MAc, Sigma-Aldrich, St. Louis, MO, USA) and α-picoline (catalyst, Fluka, Taufkirchen, Germany) according to the procedure reported in [6,14]. The substances 2-hydroxyethyl methacrylate (HEMA, Sigma-Aldrich), 2,2,4(2,4,4)-trimethylhexyl-1,6-diisocyanate (TMDI, Sigma-Aldrich), isophorone diisocyanate (IPDI, Sigma-Aldrich), 4,4’-methylenebis(cyclohexyl isocyanate) (CHMDI, Sigma-Aldrich), 2,4-toluene diisocyanate (TDI, Sigma-Aldrich) and triethylene glycol dimethacrylate (TEGDMA, Sigma-Aldrich) were used as received.

The structure of all the monomers was confirmed in 1H NMR experiments (300 MHz spectrometer, Varian UNITY/INOVA, Palo Alto, CA, USA), performed in CDCl_3_ solution, using tetramethylsilane (TMS) as a reference (Sigma-Aldrich).

### 3.2. Curing Procedure

The monomers were mixed with: 0.4 wt% of camphorquinone (CQ, Sigma-Aldrich)—the photosensitizer—and 1 wt% of N,N-dimethylaminoethyl methacrylate (DMAEMA, Sigma-Aldrich)—the reducing agent—and after vigorous stirring, poured into moulds—Petri dishes (120 mm in diameter and 4 mm thick). The samples were covered with PET film in order to reduce the effects of oxygen inhibition and then irradiated, at room temperature, for thirty minutes. Photopolymerization was initiated with a high pressure mercury vapor lamp (FAMED-1, model L-6/58, Lodz, Poland, power 375 W [6,14]), emitting UV/VIS light, where CQ absorbs in the 420–500 nm range [3].

### 3.3. FTIR Spectroscopy

The degree of conversion (*DC*) in studied polymers was determined by using FTIR spectrophotometer (Bio-Rad Laboratories, model FTS 175C, Richmond, CA, USA). The spectra of the monomers and their polymers were recorded with 128 scans at a resolution of 1 cm^−1^. The absorption intensity of selected peaks was measured in the 1800–1500 cm^−1^ region as a baseline [8]. The monomer samples were tested as very thin films on potassium bromide pellets. The cured samples were pulverized into fine powder, of a particle diameter less than 24 μm, and analyzed as pellets with potassium bromide. The *DC* was calculated from the decrease of absorption band at 1637 cm^−1^, referring to the C=C stretching vibration (*A*_C=C_), in relation to the peak at 1718 cm^−1^, assigned to the C=O stretching vibrations (*A*_C=O_):

(1) $$DC(\%)=(1-\frac{{\left(A_{C=C}/A_{C=O}\right)}_{polymer}}{{\left(A_{C=C}/A_{C=O}\right)}_{monomer}})\times 100$$

### 3.4. Mechanical Properties

#### 3.4.1. Flexural Properties

The flexural modulus (*E*) and the flexural strength (σ) were determined in accordance with ISO 178 in three-point bending tests, using a universal testing machine (INSTRON, model TT-CM, Norwood, MA, USA) [23]. Rectangular samples of UDMA polymers (length × width × thickness: 80 mm × 10 mm × 4 mm) were cut from moulds, prepared as previously mentioned. The *E* and the σ were calculated following these equations, respectively:

(2) $$E(MPa)=\frac{P_{1}l^{3}}{4bd^{3}\delta }$$

and

(3) $$\sigma \;(MPa)=\frac{3Pl}{2bd^{2}}$$

where, *P* is the maximum load; *P*_1_–the load at a selected point of the elastic region of the stress-strain plot; *l*–the distance between supports; *b*–the width of the specimen; *d*–the thickness of the specimen; δ–the deflection of the specimen at *P*_1_.

#### 3.4.2. Hardness

The ball indentation hardness (*HB*) was determined according to ISO 2039-1, on disc-like test specimens (diameter × thickness: 120 mm × 4 mm), using VEB Werkstoffprüfmaschinen apparatus (Leipzig, Germany) [24]. *HB* was calculated according to:

(4) $$HB\;(MPa)=\frac{F_{m}(\frac{0.21}{h-h_{r}+0.21})}{\text{π}dh_{r}}$$

where, *F_r_* is the reduced test load; *h_r_*, the reduced depth of impression (*h_r_* = 0.25 mm); *d*, the diameter of the ball indenter (*d* = 5 mm); *F_m_*, the test load on the indenter (*F_m_* = 490 N); *h*, the depth of impression.

#### 3.4.3. Impact Strength

The impact strength was determined in accordance with PN-68/C-89028 using VEB Werkstoffprüfmaschinen Dynstat apparatus [25]. Rectangular specimens of each polymer (length × width × thickness: 15 mm × 10 mm × 4 mm) were cut from moulds, prepared in the same way as mentioned above. The impact strength (*a_n_*) was calculated according to the formula:

(5) $$a_{n}\;(\text{kJ}/m^{2})=\frac{A_{n}}{bd}$$

where, *A_n_* is the impact energy required to cause a material to fracture; *b*, *d*–respectively, the width and the thickness of the specimen.

### 3.5. Scanning Electron Microscopy

Morphology investigations were performed on fractured surfaces of the cured materials with the Hitachi TM-3000 Scanning Electron Microscope, Tokyo, Japan (SEM). The sample surfaces, before the observations, were sputter coated with gold.

## 4. Conclusions

The mechanical properties of the urethane-dimethacrylate homopolymers and their copolymers with Bis-GMA and TEGDMA were shown to be dependent on the monomer elasticity, the concentration of double bonds, the strength of hydrogen bonds, the degree of conversion in polymers as well as to their morphology. In general, the UDMA homopolymers had the appropriate hardness, flexural modulus, and flexural strength for dental applications. High brittleness appeared to be their weakest property, especially for the polymer produced from short and stiff HEMA/IPDI. The reason for this high brittleness might be a high structural heterogeneity, resulting from the microgel agglomerate formation, as well as insufficient strength of hydrogen bonds between urethane groups. Thereby well-defined, microgel agglomerates could not be sufficiently bonded to withstand high impact energy. Monomers not involved in hydrogen bonding, such as TEGDMA, form smoother polymer morphology, composed of smaller agglomerates with higher crosslink density. Thus the degree of conversion in the matrix, surrounding the microgel agglomerates, is probably higher, causing an increase in impact strength. Bis-GMA had the highest structural heterogeneity, which was revealed by having the sharpest SEM fracture pattern. However, hydrogen bonds between -OH groups are the strongest of the studied systems, which caused higher impact resistance of Bis-GMA than that of HEMA/IPDI polymers. The improvement of impact strength among UDMA homopolymers was achieved by using DEGMMA/IPDI, DEGMMA/TDI and TEGMMA/TDI monomers having longer wings and asymmetrically substituted cycloaliphatic and aromatic diisocyanates. The TEGMMA, having three oxyethylene units, merely resulted in a satisfactory increase in hardness. The methylene dicyclohexylene core in the DEGMMA/CHMDI monomer did not improve the homopolymer impact resistance at all.

The copolymerization of UDMA always resulted in improved degree of conversion, impact resistance, and hardness. The increase in the flexural strength was only observed for copolymers of HEMA/IPDI with TEGDMA. Flexural strength of the remaining copolymers and flexural modulus of all copolymers decreased in relation to the corresponding UDMA homopolymers. However, these values might be satisfactory for dental applications and are usually higher than those of popular dental compositions: Bis-GMA–TEGDMA (60:40) and HEMA/TMDI–Bis-GMA–TEGDMA.

None of the alternative UDMA monomers could be dedicated for use as a single dental composite resin. However, their copolymerization had a positive impact on the structure and properties of the polymer network. Comparing the HEMA/IPDI–TEGDMA (80:20) copolymer to the Bis-GMA–TEGDMA (60:40) copolymer, which is often used as a dental composite matrix, the first one had a higher degree of conversion, hardness, flexural modulus, and flexural strength. Although the impact resistance of HEMA/IPDI–TEGDMA (80:20) copolymer was lower than that of Bis-GMA–TEGDMA (60:40) and HEMA/TMDI–Bis-GMA–TEGDMA copolymers, it was higher than the impact resistance of the HEMA/TMDI homopolymer, which is sometimes used alone in dental composites.

The copolymer of DEGMMA/IPDI–Bis-GMA–TEGDMA had the finest mechanical performance and could be suggested for future applications in dental composite materials. This polymer combines high hardness, flexural strength, and impact strength. Its flexural modulus is appropriately high, being lower than that of the Bis-GMA–TEGDMA (60:40) copolymer, but higher than the modulus of the HEMA/TMDI–Bis-GMA–TEGDMA copolymer.
